# Clinical efficacy and health implications of inconsistency in different production batches of antimycotic drugs in a developing country

**DOI:** 10.4103/0975-7406.76501

**Published:** 2011

**Authors:** Adenike A. O. Ogunshe, Adedayo A. Adepoju, Modupe E. Oladimeji

**Affiliations:** Applied Microbiology and Infectious Diseases, Department of Microbiology, University of Ibadan, Ibadan, Nigeria; 1 Department of Statistics, University of Ibadan, Nigeria, Department of Mathematics, University of Mines and Technology, Tarkwa, Ghana; 2 Microbiology and Virology Unit, Laboratory Technology Training School, University of Ibadan, Ibadan, Nigeria

**Keywords:** Antifungal agents, candidosis, clinical efficacy, production batch, public health

## Abstract

**Objective:**

This study aimed at evaluating the *in vitro* efficacy and health implications of inconsistencies in different production batches of antimycotic drugs.

**Materials and Methods:**

*in vitro* susceptibility profiles of 36 *Candida* spp. – *C. albicans* (19.4%), *C. glabrata* (30.6%), *C. tropicalis* (33.3%), and *C. pseudotropicalis* (16.7%) – obtained from human endocervical and high vaginal swabs (ECS/HVS) to two different batches (B1 and B2) of six antimycotic drugs (clotrimazole, doxycycline, iconazole, itraconazole, metronidazole and nystatin) was determined using modified agar well-diffusion method.

**Results:**

None of the *Candida* strains had entirely the same (100%) susceptibility / resistance profiles in both batches of corresponding antimycotic drugs; while, different multiple antifungal susceptibility (MAS) rates were also recorded in batches 1 and 2 for corresponding antifungals. Only 14.3%, 27.3%, 16.7-33.3%, and 8.3-25.0% of *C. albicans*, *C. glabrata*, *C. pseudotropicalis*, and *C. tropicalis* strains, respectively, had similar susceptibility/resistance profiles toward coressponding antifungal agents in both batches; while up to 57.1% of *C. albicans*, 45.5% of *C. glabrata*, 66.7% of *C. pseudotropicalis*, and 50.0% of *C. tropicalis* strains were susceptible to one batch of antifungals but resistant to corresponding antifungals in the second batch. As high as 71.4% (*C. albicans*), 73.0% (*C. glabrata*), 50.0% (*C. pseudotropicalis*), and 66.74% (*C. tropicalis*) strains had differences of ≥ 10.0 mm among corresponding antimycotic agents.

**Conclusions:**

*Candida* strains exhibited different *in vitro* susceptibility / resistance patterns toward two batches of corresponding antimycotic agents, which has clinical implications on the efficacy of the drugs and treatment of patients. The findings of the present study will be of benefit in providing additional information in support of submission of drugs for registration to appropriate regulatory agencies.

The incidence of yeast infections has increased in recentdecades,[1] while invasive infections by opportunistic *Candida* spp. have also been reported to have significant impact on human morbidity and mortality.[2] *Candida*, once considered as a minor pathogen,is now among the most commonly cultured pathogenic microorganisms, even in intensivecare units (ICU),[3–6] while vulvovaginal candidosis, which affects all strata of the society, has remained a common problem worldwide.[7] However, in a developing country like Nigeria, apart from the addresses on the packages of clinical drugs in pharmacies, the sources of most of the drugs cannot be fully authenticated or ascertained. Even, in spite of the massive activities by the National Agency for Food, Drugs Administration and Control (NAFDAC) against production and importation of adulterated and substandard drugs into the country, fake drugs are still reported on regular basis.

A counterfeit formulation is one that is deliberately and fraudulently mislabeled with respect to identification and/or source. Counterfeiting can apply to both branded and generic products and counterfeits may include products with the incorrect ingredients or with the wrong ingredients, without active ingredients, with insufficient active ingredient, or with fake packaging,[8] and it is known that drug quality in public and private outlets may be problematic. A previous study in Nigeria, which assessed the quality of drugs from retail outlets and pharmacies, attributd the problems of counterfeit drugs to lack of quality control in manufacture, as well as degradation during storage.[9] There is also a little existing knowledge about actual quality of drugs provided by different providers in Nigeria and in many sub-Saharan African (SSA) countries.

A search of the medical literature yielded only 43 primary published research reports concerning counterfeit drugs in the world,[10] while failing products more often originated or were claimed to originate from poorer parts of the world with weaker regulatory systems.[11] Over the past decade, the massive public health problem of counterfeit and substandard drugs has become more manifest, leading to serious clinical consequences on patients, such as increased morbidity, mortality, and drug resistance, which contributes to spurious reporting of resistance and toxicity, as well as loss of confidence in the healthcare systems.[10] Other studies looking at a broader range of diseases in Nigeria found widespread inappropriate drug use, low quality of treatment and ineffective regulations.[12–14]

Quick results of *in vitro* susceptibility testing of *Candida* spp. to the common antifungal agents are desirable,[15] but usage of inconsistent batches of antimycotics, which can give varying results during treatments, calls for general concerns. The aim of this study is, therefore, to compare the susceptibility patterns of vulvovaginal candidiasis-associated *Candida* strains isolated from ECS and HVS clinical specimens to two different batches of the most-available antifungal agents in the country.

## Materials and Methods

### Identification of yeast isolates

A total of 36 strains of *Candida* isolated from high vaginal swabs (HVS) and endocervical swabs (ECS), which were obtained by clinical routine from patients who presented for candidosis and who had not been on antimycotic therapy in about 6 months prior to time of collection, were obtained from the culture collections of the Department of Medical Microbiology and Parasitology, University College Hospital, Ibadan, Nigeria. The *Candida* strains were sub-cultured by streaking on Sabouraud dextrose agar (SDA) (Lab M, England) plates and incubated at 32°C for 24-48 hours until assure purity, and characterized according to their colonial characteristics on CHROM-agar, microscopic morphology, as well as biochemical tests, including assimilation of sugars- cellobiose, dextrose, dulcitol, fructose, galactose, inositol, lactose, maltose, mannitol, mellibiose, rhamnose, saccharose, sorbitol, sucrose, xylose. The identification of the *Candida* strains was based on standard phenotypic taxonomic tools and clinical practices as previously described.[16,17] In addition, fresh wet mount examinations (wet preparations) and germinal tube assay were also performed on the yeast strains, and pure, identified strains were kept in triplicates on SDA agar slants at 4°C as bench and stock cultures.

*In vitro* antimycotic susceptibility testing . *In vitro* susceptibilities / resistance to commonly available antimycotic agents in Nigeria [the imidazoles-mycoten tablets/cream, canesten tablets/cream (i.e., clotrimazoles); tetradox (doxycycline); the polyenes-mycostatine (nystatin), and the metronidazole- flagyl] were determined on SDA at 35°C after 24 and 48 hours ofincubation, using the modified method[18] of Tagg *et al.*[19] The concentration of the inoculum suspensions of the test *Candida* isolates were between 1.6 and 2.4 × 10^3^ cells ml^-1^.

### Statistical analysis

Tests of hypothesis using chi-square and ANOVA were carried out to show if there exists a significant difference between the two batches of antimycotic agents (B1 and B2).[20,21]

## Results

The *Candida* spp. isolated from clinical specimens (CV/HVS) were characterized in this study as *C. albicans* (19.4%), *C. glabrata* (30.6%), *C. tropicalis* (33.3%), and *C. pseudotropicalis* (16.7%). None of the *Candida* strains had 100% susceptibility profiles toward all the antimycotic agents in both batches. Varying multiple antifungal susceptibility (MAS) rates of 14.3-100%/85.7-100%; 28.6-100%/28.6-100%; 28.6-100%/14.3-100%, and 42.9-85.7%/14.3-100% were recorded in batches 1 and 2 among the *C. albicans*, *C. glabrata*, *C. tropicalis*, and *C. pseudotropicalis* strains, respectively, but wider zones of inhibition were recorded in batch 2 antifungal drugs [Tables 1–4].

**Table 1:** Phenotypic antimycotic susceptibility/resistance profiles of *Candida albicans* strains associated with candidiasis to two batches of same antimycotic drugs

Candida strains	Antimycotic agents (μg ml^-1^)								
Total (7)	B1	AF1	AF2	AF3	AF4	AF6	AF8	AF9	%MAS
	B2	AM2	AM9	AM5	AM8	AM1	AM4	AM7	
*C. albicans* C2	B1	R	R	R	R	R	20.0	R	1 (14.3)
	B2	20.0	25.0	18.0	10.0	24.0	30.0	24.0	7 (100)
*C. albicans* C29	B1	10.0	10.0	20.0	10.0	10.0	20.0	R	6 (85.7)
	B2	25.0	26.0	28.0	24.0	30.0	32.0	26.0	7 (100)
*C. albicans* C51	B1	10.0	R	10.0	R	20.0	20.0	10.0	5 (71.4)
	B2	R	28.0	20.0	25.0	36.0	30.0	28.0	6 (85.7)
*C. albicans* C23	B1	10.0	20.0	10.0	20.0	10.0	20.0	R	6 (85.7)
	B2	14.0	18.0	25.0	14.0	R	20.0	30.0	6 (85.7)
*C. albicans* 2C2	B1	R	20.0	20.0	R	20.0	25.0	20.0	5 (71.4)
	B2	28.0	26.0	30.0	26.0	32.0	28.0	28.0	7 (100)
*C. albicans* GC2	B1	10.0	10.0	20.0	10.0	20.0	20.0	R	6 (85.7)
	B2	30.0	24.0	30.0	28.0	30.0	35.0	16.0	7 (100)
*C. albicans* 6C1	B1	10.0	10.0	10.0	10.0	10.0	10.0	10.0	7 (100)
	B2	30.0	24.0	28.0	24.0	28.0	35.0	20.0	7 (100)
Total/(%) susceptibility	B1	5 (71.4)	5 (71.4)	6 (85.7)	4 (57.1)	6 (85.7)	7 (100)	3 (42.9)	
	B2	6 (85.7)	7 (100)	7 (100)	7 (100)	6 (85.7)	7 (100)	7 (100)	
S/R		3 (42.9)	2 (28.6)	1 (14.3)	3 (42.9)	2 (28.6)	- (0.0)	4 (57.1)	
≤ 5.0 mm		1 (14.3)	1 (14.3)	- (0.0)	- (0.0)	- (0.0)	1 (14.3)	- (0.0)	
≥10.0 mm		3 (42.9)	3 (42.9)	5 (71.4)	3 (42.9)	5 (71.4)	5 (71.4)	2 (28.6)
*		- (0.0)	- (0.0)	- (0.0)	- (0.0)	- (0.0)	1 (14.3)	- (0.0)	

* same values in corresponding antimycotics of both batches. Values of zones of inhibition are means of duplicates

*P* = 0.016646. B1, Batch 1; B2, Batch 2; AF1/AM2, mycoten tablets; AF2/AM9, mycoten cream; AF3/AM5, canesten tablets (clotrimoxazole); AF4/AM8, canesten cream (clotrimoxazole); AF6/AM1, tetradox (doxycycline); AF8/AM4, mycostatine (nystatin); AF9 AM7, flagyl (metronidazole). S/R, corresponding antimycotics susceptible in one batch but resistant in the other batch; ≤5.0 mm, corresponding antimycotics having zones of inhibition differences of ≤5.0 mm in diameter; ≥10.0 mm, corresponding antimycotics having zones of inhibition differences of ≥10.0 mm in diameter;

**Table 2:** Phenotypic antimycotic susceptibility/resistance profiles of *Candida glabrata* strains associated with candidiasis to two batches of same antimycotic drugs

*Candida* strains	Antimycotic agents (μg ml^-1^)								
Total (11)	B1	AF1	AF2	AF3	AF4	AF6	AF8	AF9	%MAS
	B2	AM2	AM9	AM5	AM8	AM1	AM4	AM7	
*C. glabrata* C3	B1	R	R	R	R	24.0	20.0	R	2 (28.6)
	B2	R	R	R	R	20.0	32.0	R	2 (28.6)
*C. glabrata* C6	B1	10.0	10.0	20.0	10.0	15.0	10.0	R	6 (85.7)
	B2	35.0	R	R	24.0	32.0	36.0	28.0	5 (71.4)
*C. glabrata* C12	B1	R	20.0	R	10.0	R	20.0	R	3 (42.9)
	B2	R	R	R	R	15.0	28.0	R	2 (28.6)
*C. glabrata* C27	B1	R	R	20.0	20.0	20.0	25.0	20.0	5 (71.4)
	B2	29.0	32.0	28.0	30.0	35.0	30.0	28.0	7 (100)
*C. glabrata* 34	B1	25.0	25.0	20.0	25.0	20.0	20.0	20.0	7 (100)
	B2	28.0	20.0	24.0	30.0	30.0	35.0	28.0	7 (100)
*C. glabrata* 42	B1	20.0	20.0	20.0	20.0	20.0	20.0	R	6 (85.7)
	B2	R	R	R	R	18.0	30.0	R	2 (28.6)
*C. glabrata* 43	B1	15.0	15.0	15.0	15.0	25.0	20.0	15.0	7 (100)
	B2	28.0	34.0	28.0	30.0	35.0	24.0	38.0	7 (100)
*C. glabrata* 61	B1	10.0	10.0	10.0	10.0	R	20.0	R	5 (71.4)
	B2	26.0	27.0	30.0	26.0	35.0	35.0	24.0	7 (100)
*C. glabrata* 1TC	B1	20.0	25.0	20.0	20.0	20.0	20.0	R	6 (85.7)
	B2	26.0	24.0	28.0	22.0	30.0	34.0	26.0	7 (100)
*C. glabrata* BC2	B1	20.0	R	10.0	10.0	10.0	20.0	R	5 (71.4)
	B2	30.0	27.0	30.0	28.0	34.0	30.0	24.0	7 (100)
*C. glabrata* 4C1	B1	R	10.0	R	10.0	R	20.0	R	3 (42.9)
	B2	32.0	28.0	26.0	26.0	30.0	32.0	24.0	7 (100)
Total/(%) Susceptibility	B1	7 (63.6)	8 (72.7)	8 (72.7)	10 (90.9)	8 (72.7)	11 (100)	3 (27.3)	
	B2	8 (72.7)	7 (63.6)	7 (63.6)	8 (72.7)	11 (100)	11 (100)	8 (72.7)	
S/R		3 (27.3)	5 (45.5)	3 (27.3)	2 (18.1)	3 (27.3)	- (0.0)	5 (45.5)	
≤5.0 mm		1 (9.1)	2 (18.2)	1 (9.1)	2 (18.1)	2 (18.1)	2 (18.1)	- (0.0)	
≥10.0 mm		4 (36.4)	3 (27.3)	3 (27.3)	6 (54.5)	6 (54.5)	8 (72.7)	1 (9.1)	
*		2 (18.1)	1 (9.1)	2 (18.1)	1 (9.1)	- (0.0)	1 (14.1)	3 (27.3)	

* same values in corresponding antimycotics of both batches. Values of zones of inhibition are means of duplicates

*P* = 0.238954. B1, Batch 1; B2, Batch 2; AF1/AM2, mycoten tablets; AF2/AM9, mycoten cream; AF3/AM5, canesten tablets (clotrimoxazole); AF4/AM8, canesten cream (clotrimoxazole); AF6/AM1, tetradox (doxycycline); AF8/AM4, mycostatine (nystatin); AF9 AM7, flagyl (metronidazole). S/R, corresponding antimycotics susceptible in one batch but resistant in the other batch; ≤5.0 mm, corresponding antimycotics having zones of inhibition differences of ≤5.0 mm in diameter; ≥ 10.0 mm, corresponding antimycotics having zones of inhibition differences of ≥ 10.0 mm in diameter;

**Table 3:** Phenotypic antimycotic susceptibility/resistance profiles of *Candida pseudotropicalis* strains associated with candidiasis to two batches of same antimycotic drugs

*Candida* strains	Antimycotic agents (μg ml^-1^)								
Total (6)	B1	AF1	AF2	AF3	AF4	AF6	AF8	AF9	%MAS
	B2	AM2	AM9	AM5	AM8	AM1	AM4	AM7	
*C. pseudotropicalis* 16	B1	25.0	30.0	25.0	30.0	15.0	15.0	R	6 (85.7)
	B2	34.0	32.0	30.0	24.0	28.0	34.0	30.0	7 (100)
*C. pseudotropicalis* 25	B1	10.0	20.0	10.0	20.0	10.0	20.0	R	6 (85.7)
	B2	28.0	24.0	30.0	26.0	26.0	32.0	30.0	7 (100)
*C. pseudotropicalis* 48	B1	R	20.0	20.0	R	R	R	10.0	3 (42.9)
	B2	R	R	R	R	R	22.0	R	1 (14.3)
*C. pseudotropicalis* 65	B1	10.0	20.0	10.0	20.0	10.0	20.0	R	6 (85.7)
	B2	20.0	25.0	R	R	15.0	25.0	R	4 (57.1)
*C. pseudotropicalis* X7C	B1	10.0	10.0	10.0	10.0	10.0	20.0	R	6 (85.7)
	B2	R	R	R	R	32.0	30.0	R	2 (.27.6)
*C. pseudotropicalis* 6C2	B1	R	R	R	R	R	R	R	- (0.0)
	B2	26.0	28.0	24.0	28.0	34.0	32.0	28.0	7 (100)
Total/(%) Susceptibility	B1	4 (66.6)	5 (83.3)	5 (83.3)	4 (66.6)	4 (66.6)	4 (66.6)	1 (16.7)
	B2	4 (66.6)	4 (66.6)	3 (50.0)	3 (50.0)	5 (83.3)	6 (100)	3 (50.0)	
S/R		2 (33.3)	3 (50.0)	4 (66.7)	3 (50.0)	1 (16.7)	2 (33.3)	4 (66.7)	
≤5.0 mm		- (0.0)	3 (50.0)	1 (16.7)	- (0.0)	1 (16.7)	1 (16.7)	- (0.0)	
≥10.0 mm		2 (33.3)	- (00.0)	1 (16.7)	- (00.0)	3 (50.0)	3 (50.0)	- (0.0)	
*		1 (16.7)	- (0.0)	- (0.0)	1 (16.7)	1 (16.7)	1 (16.7)	2 (33.3)	

* same values in corresponding antimycotics of both batches. Values of zones of inhibition are means of duplicates

*P* =0.372246. B1, Batch 1; B2, Batch 2; AF1/AM2, mycoten tablets; AF2/AM9, mycoten cream; AF3/AM5, canesten tablets (clotrimoxazole); AF4/AM8, canesten cream (clotrimoxazole); AF6/AM1, tetradox (doxycycline); AF8/AM4, mycostatine (nystatin); AF9 AM7, flagyl (metronidazole). S/R, corresponding antimycotics susceptible in one batch but resistant in the other batch; ≤5.0 mm, corresponding antimycotics having zones of inhibition differences of ≤5.0 mm in diameter; ≥10.0 mm, corresponding antimycotics having zones of inhibition differences of ≥10.0 mm in diameter;

**Table 4:** Phenotypic antimycotic susceptibility/resistance profiles of Candida tropicalis strains associated with candidiasis to two batches of same antimycotic drugs

*Candida* strains	Antimycotic agents (μg ml^-1^)								
Total (12)	B1	AF1	AF2	AF3	AF4	AF6	AF8	AF9	%MAS
	B2	AM2	AM9	AM5	AM8	AM1	AM4	AM7	
*C. tropicalis* C8	B1	R	R	R	20.0	R	20.0	R	2 (28.6)
	B2	R	R	R	R	28.0	35.0	30.0	3 (42.9)
*C. tropicalis* C9	B1	R	R	20.0	R	20.0	10.0	R	3 (42.9)
	B2	28.0	30.0	28.0	24.0	28.0	34.0	26.0	7 (100)
*C. tropicalis* C14	B1	25.0	20.0	R	25.0	10.0	10.0	R	5 (71.4)
	B2	30.0	28.0	30.0	26.0	30.0	30.0	28.0	7 (100)
*C. tropicalis* C20	B1	R	20.0	R	R	R	20.0	R	2 (28.6)
	B2	R	R	R	R	30.0	23.0	20.0	3 (42.9)
*C. tropicalis* 26	B1	10.0	R	10.0	20.0	R	20.0	R	4 (57.1)
	B2	30.0	28.0	26.0	28.0	32.0	38.0	18.0	7 (100)
*C. tropicalis* 40	B1	30.0	30.0	30.0	30.0	30.0	30.0	30.0	7 (100)
	B2	26.0	28.0	30.0	22.0	30.0	34.0	24.0	7 (100)
*C. tropicalis* 53	B1	10.0	10.0	20.0	10.0	10.0	25.0	R	6 (85.7)
	B2	R	R	15.0	R	20.0	30.0	R	3 (42.9)
*C. tropicalis* 10C	B1	10.0	10.0	10.0	20.0	10.0	20.0	R	6 (85.7)
	B2	R	R	R	R	R	25.0	R	1 (14.3)
*C. tropicalis* 2TC	B1	10.0	R	10.0	10.0	10.0	20.0	R	5 (71.4)
	B2	R	28.0	R	R	R	30.0	R	2 (28.6)
*C. tropicalis* HC	B1	10.0	10.0	10.0	10.0	10.0	20.0	10.0	7 (100)
	B2	28.0	26.0	26.0	28.0	30.0	32.0	18.0	7 (100)
*C. tropicalis* 6C	B1	10.0	10.0	10.0	10.0	10.0	20.0	R	6 (85.7)
	B2	30.0	24.0	28.0	28.0	32.0	31.0	27.0	7 (100)
*C. tropicalis* 9C	B1	R	10.0	10.0	15.0	R	20.0	10.0	5 (71.4)
	B2	30.0	24.0	26.0	28.0	32.0	30.0	26.0	7 (100)
Total/(%) Susceptibility	B1	8 (66.6)	8 (66.6)	4 (66.6)	10 (83.3)	8 (66.6)	12 (100)	3 (25.0)	
	B2	7 (58.4)	8 (66.6)	8 (66.6)	7 (58.4)	10 (83.3)	12 (100)	9 (75.0)	
R/S		5 (41.6)	6 (50.0)	3 (25.0)	5 (41.6)	6 (50.0)	- (0.0)	6 (50.0)
≤5.0 mm		2 (16.7)	1 (8.3)	2 (16.7)	1 (8.3)	- (0.0)	4 (33.3)	- (0.0)	
≥10.0 mm		3 (25.0)	3 (25.0)	4 (33.3)	3 (25.0)	4 (33.3)	8 (66.7)	1 (8.3)	
*		2 (16.7)	1 (8.3)	3 (25.0)	1 (8.3)	1 (8.3)	- (0.0)	3 (25.0)	

* same values in corresponding antimycotics of both batches. Values of zones of inhibition are means of duplicates

*P* = 0.409089. B1, Batch 1; B2, Batch 2; AF1/AM2, mycoten tablets; AF2/AM9, mycoten cream; AF3/AM5, canesten tablets (clotrimoxazole); AF4/AM8, canesten cream (clotrimoxazole); AF6/AM1, tetradox (doxycycline); AF8/AM4, mycostatine (nystatin); AF9 AM7, flagyl (metronidazole). S/R, corresponding antimycotics susceptible in one batch but resistant in the other batch; ≤5.0 mm, corresponding antimycotics having zones of inhibition differences of ≤5.0 mm in diameter; ≥10.0 mm, corresponding antimycotics having zones of inhibition differences of ≥10.0 mm in diameter;

Among the *C. albicans*, just 14.3% of the strains had same susceptibility/resistance profiles toward the same test antifungal agents in both batches 1 and 2, while up to 57.1% of the strains were susceptible in the first batch but resistant to corresponding antifungals in the second batch. Between 28.6% and 71.4% of the *C. albicans* had difference of ≥10.0 mm (zones of inhibition) as the recorded values in corresponding antimycotic agents [Table 1].

Only a maximum of 27.3% of *C. glabrata* strains had same susceptibility/resistance profiles, with as high as about 73.0% of the strains having differences of ≥10.0 mm (zones of inhibition) among corresponding antimycotic agents, while between 18.1% and 45.5% were susceptible in a batch and resistant to corresponding antifungals in the second batch [Table 2].

As shown in Table 3, as low as 16.7-33.3% of the *C. pseudotropicalis* strains had same susceptibility/resistance profiles, while about 50.0% of the strains had differences of ≥10.0 mm (zones of inhibition) among corresponding antimycotic agents, with as high as 66.7% of the strains being susceptible in a batch but resistant to corresponding antifungals in the second batch. *C. pseudotropicalis* 6C2 was resistant against all test antifungal agents in the batch 1 but susceptible towards all the test antifungals in batch 2.

Table 4 shows that between 8.3% and 25.0% of the *C. tropicalis* strains had same susceptibility/resistance profiles toward the test antifungal agents in both batches, while up to 50.0% of the strains were susceptible in one batch but resistant to corresponding antifungals in the second batch. As high as 66.74% of the *Candida* strains had differences of ≥10.0 mm (zones of inhibition) toward the corresponding antimycotic agents in the other batch.

Raw nonstatistical data indicated that most of the *Candida* strains were different in their susceptibility/resistance profiles toward the same antimycotic agents in the two batches, i.e., the *in vitro* susceptibility tests on the *Candida* strains revealed that the inhibitory activities of the two batches of antimycotic agents were significantly different from each other. Some of the *Candida* strains like *C. glabrata* C27, C43, C61, 1TC, BC2, 4C1; *C. tropicalis* C9, C14, C26, C53, 10C, 2TC, HC, 6C; *C. pseudotropicalis* X7C, 6C2 were found to have well-defined differences in their susceptibility profiles toward the two batches of same antifungal agents, meaning that the two antimycotic agents in batches B1 and B2 had different inhibitory effects or potency on the *Candida* strains.

Relatively higher susceptibility rates were recorded among the antifungals in batch B2 compared to batch B1 - *C. albicans* (95.9%; 73.5%), *C. glabrata* (77.9%; 71.4%), *C. tropicalis* (66.7%; 64.3%), and *C. pseudotropicalis* (72.6%; 63.1%); while the statistical results indicated the recorded susceptibility values as *C. albicans* (p=0.016646), *C. glabrata* (0.238954), *C. tropicalis* (0.372246), and *C. pseudotropicalis* (0.409089), respectively [Tables 1–4].

## Discussion

Vaginal discharge is the symptom that most often prompts a woman to consult a physician in order to determine the presence of an infection, while diagnosis is usually based on evaluation of the vaginal ecosystem and demonstration of the presence of the suspected microorganisms.[22] In the study of Wathne *et al*[23] and the review of Sobel,[24] on the epidemiology, diagnosis, and therapy of vaginitis, it was reported that vulvovaginal symptoms are extremely common and can cause extreme distress for some patients, especially those with recurrent symptoms.[25] Women, therefore, often seek medical care for vaginal complaints.[26] *Candida* infectious complications in pregnancy and delivery are still very serious problems in obstetrical, gynecological, and neonatological practices, and the presence of vaginal infections during pregnancy has also been linked to low birth weight and obstetric disorders.[22,27] Similarly, *C. albicans*, *C. glabrata*, *C. tropicalis*, and *C. pseudotropicalis*, which are among the most implicated species in vulvovaginal *Candida*sis were also recovered from symptomatic females in this study. It is, therefore, very important that vulvovaginal *Candida*sis must be promptly treated.

Several antifungal agents are available for the treatment of Candidasis,[28] but there have been reports of antagonism between antifungal compounds and isolates of *Candida* spp.[29–31] The *in vitro* activities of antifungal agents, however, varied among various studies,[32–35] with differing spectra of activities against antifungal agents, while *in vitro* testing has similarly revealed that there are clear differences among the various non-albicans *Candida* (NAC) and *C. albicans* in their susceptibility to specific antifungal drugs. It is also generally believed that there is a significant increase in the resistance of *Candida* spp. toward antifungal agents in recent times.[35,36] It was, however, observed in the current study that according to the overall results obtained, most of the *Candida* strains were susceptible to the test antifungal agents, especially mycostatine, tetradox, canesten cream, and mycoten tablet, which is in accordance with some previous studies that recorded relatively higher susceptibility rates toward certain antifungal agents by some *Candida* strains implicated in vulvovaginal Candidasis.[35–39]

The relatively high differences in the susceptibility/resistance result patterns obtained from the two batches of corresponding antifungal drugs in this study are of serious significance and also corroborated the hazardous effect of inconsistent drug production under different production batches, which must be taken into consideration when screening and choosingantifungal agents for fungal therapy. In bioequivalence studies, the goal of testing is to determine if the drugs are functionally equivalent, due to the fact that a drug may be chemically equivalent but not clinically equivalent.[40] As an example, routine antibiotic susceptibility testing has been advocated as an essential selection criterion for potential probiotic Candidates but in a previous study,.[41] while determining the phenotypic antibiotic susceptibility of 54 potential probiotic Candidates to the same antibiotics of different production batches, it was found that the overall percentage differences among the probiotic Candidates to the same test antibiotics of different production batches, manufactured by the same company, were between 53.9% and 76.5%. The implication is that if one batch of antibiotics had been used, some potential probiotic Candidates would have been eliminated by the resistance selection criterion.

Two drugs are considered pharmaceutical equivalents when they contain the same chemically active ingredient(s) and are identical in dosage form and strength,[42] but pharmaceutical equivalence may be affected by variations in inert ingredients, such as production of ingredients that vary in quality, and by batch and manufacturing methods.[43] Another factor which affects generic quality is the international buyouts and diversification, which allows the combination of questionable ingredients into generic production.[44] Most of the times, once a drug has been approved by the regulating bodies like FDA or NAFDAC, manufacturers sometimes make changes to the formulations, which were originally submitted for screening.[43,44] Although drug quality is currently receiving renewed international attention[45] but in spite of an increase in public awareness of the existence of counterfeit and substandard drugs over the past decade,[46] it is quite unfortunate that the menace of counterfeit and substandard drugs is being increasingly reported in developing countrieslike Nigeria due to ineffective drug regulations.[10,47]

There is growing universal concern regarding counterfeit medications, and in particular, counterfeit antimicrobial drugs are a threatto public health with many devastating consequences for patients, such as, increased mortality and morbidity, and emergence of drug resistance. In addition, physicians treating these patients lose their confidencein the medications used due to report of high levels of resistance.[48] Usually, the way products are manufactured depends on the quantity required but the inconsistencies in activities associated with batch production of clinical drugs may be due to the fact that it is not a continuous production, since there is in-between stoppage and reconfiguration of equipment during production batches, especially as regards the downtime (idle time between batches) and cycle time (time between consecutive batches).[49] In the study of Khabriev and Yagudina,[50] while assessing the general state-of-the-art in the quality of domestic drugs on the Russian market, it was established that about 16.5 thousand of the drug batches rejected were recalled from the market over the period from 1994 to 2002 with the total number of rejected batches increasing from 660 in 1994 to 1107 in 2002. This is not usually the case in Nigerian situation; therefore, it is very difficult to regulate drug batches that do not meet the standard criteria.

Assessment of clinical drugs and recall of low-quality or adulterated drugs in Nigeria is minimal and not regular due to some faults in logistics, such as consideration of the production batches of drugs prior to registration by the regulating bodies. The fact that none of the *Candida* strains had entirely the same (100%) susceptibility profiles in just two batches of corresponding antimycotic drugs, while as low as 8.3-33.3% of the *Candida* strains had similar susceptibility/resistance profiles toward the test antifungal agents in both batches, confirms that there is serious clinical and health implications as regards the inconsistency in different production batches of such antimycotic drugs. Conflicting inhibitory activities of corresponding antimycotics could be a threatto public health with consequences for patients, since prescription could be made based on the assumption that inhibition by an antimycotic drug in a batch would have the same effect by corresponding drug(s) in other batch(es). Similarly, clinical implication can be deduced, in that reports of resistance/susceptibility are mostly not the same in corresponding drugs of different batches, which will ultimately lead to errors in documented findings.

It is, therefore, very important to assess the consistency of different batches of drugs with regards to potency and when understudying the susceptibility or resistance patterns of the pathogens, especially in developing countries, where most drugs in circulation are adulterated. Similarly, it is of adequate importance that every production batch of drugs in Nigeria be consistently screened by regulating bodies like NAFDAC before approval for sales and administration of such drugs. There must be complete documented investigations into the failure of drug batches, which do not meet the expected specifications. It is also important that policies are put in place to ascertain that clinical drugs are properly screened with adequate investigations into causes of manufacturing problems.
